# Site-Specific Inflammatory Signatures in Metastatic NSCLC: Insights from Routine Blood Count Parameters

**DOI:** 10.3390/medicina61091521

**Published:** 2025-08-25

**Authors:** Vlad-Norin Vornicu, Alina-Gabriela Negru, Razvan Constantin Vonica, Andrei Alexandru Cosma, Sorin Saftescu, Mihaela Maria Pasca-Fenesan, Anca Maria Cimpean

**Affiliations:** 1 Doctoral School in Medicine, “Victor Babes” University of Medicine and Pharmacy, 300041 Timisoara, Romania; vlad.vornicu@umft.ro (V.-N.V.); cosma.andrei@umft.ro (A.A.C.); mihaela.fenesan@umft.ro (M.M.P.-F.); 2 Department of Oncology, OncoHelp Hospital Timisoara, Ciprian Porumbescu Street, No. 59, 300239 Timisoara, Romania; 3 Department of Cardiology, “Victor Babes” University of Medicine and Pharmacy, 300041 Timisoara, Romania; alinanegru@umft.ro; 4 Preclinical Department, Faculty of Medicine, “Lucian Blaga” University of Sibiu, 550169 Sibiu, Romania; razvanconstantin.vonica@ulbsibiu.ro; 5 Department of Microscopic Morphology/Histology, “Victor Babes” University of Medicine and Pharmacy, 300041 Timisoara, Romania; acimpeanu@umft.ro; 6 Department of Oncology, Faculty of Medicine, “Victor Babes” University of Medicine and Pharmacy, 300041 Timisoara, Romania; 7 Center of Expertise for Rare Vascular Disease in Children, Emergency Hospital for Children Louis Turcanu, 300041 Timisoara, Romania; 8 Center of Genomic Medicine, “Victor Babes” University of Medicine and Pharmacy, 300041 Timisoara, Romania; 9 Research Center for Pharmaco-Toxicological Evaluation, “Victor Babes” University of Medicine and Pharmacy, 300041 Timisoara, Romania

**Keywords:** lung neoplasms, neoplasm metastasis, pleura, blood platelets, lymphocytes, monocytes, biomarkers, tumor, inflammation mediators

## Abstract

*Background and Objectives*: Systemic inflammatory markers from an ordinary complete blood count (CBC) may foreshadow where non-small-cell lung cancer (NSCLC) will first spread, but organ-specific signatures remain poorly defined. *Materials and Methods*: We retrospectively reviewed 302 adults (mean age 60.7 ± 13.4 years; 80.8% men) with stage IV NSCLC managed at OncoHelp Medical Center, Timișoara, between January 2022 and December 2024. Eligibility demanded a single radiologically confirmed distant site at diagnosis and pre-treatment CBC. Neutrophil-to-lymphocyte (NLR), platelet-to-lymphocyte (PLR), and lymphocyte-to-monocyte (LMR) ratios were compared across pleural (*n* = 52), bone (*n* = 86), liver (*n* = 66), and brain (*n* = 98) metastases using Kruskal–Wallis tests with Bonferroni adjustment; z-standardized logistic models identified independent predictors. *Results*: Metastases clustered most often in brain (32.5%), followed by bone (28.5%), liver (21.9%), and pleura (17.2%). Median PLR rose selectively in pleural disease (274 vs. 217–253 in other sites; *p* = 0.006). LMR fell to 2.0 in bone but climbed to 2.8 in brain lesions (*p* = 0.032 and 0.008, respectively). NLR was globally elevated (6.7–7.6), yet differed significantly only for bone and liver deposits. Logistic modeling showed that each standard-deviation rise in absolute neutrophil count quadrupled the odds of hepatic involvement (Odd Ratio (OR) 4.26; 99% Confidence inerval (CI) 2.20–6.25), monocytosis nearly doubled bone risk (OR 1.83; 1.01–3.33), while higher erythrocytes, eosinophils, and lymphocytes independently protected against pleural seeding (all *p* < 0.01). Age-stratified analysis revealed that osseous and cerebral metastases predominated in patients ≤ 50 years, whereas inflammatory indices were age-invariant. *Conclusions*: Routine CBC ratios encode distinct “inflammatory fingerprints” that mirror the first metastatic destination in NSCLC: platelets herald pleural spread, neutrophils favor liver and bone, and divergent lymphocyte–monocyte balances separate bone from brain. Although no substitute for cross-sectional imaging, these low-cost markers could refine clinical suspicion, guide targeted work-up, and illuminate the biology of organ-selective dissemination, particularly in resource-limited settings.

## 1. Introduction

Lung cancer continues to be one of the most widespread and deadly forms of cancer across the globe, with non-small-cell lung cancer (NSCLC) accounting for most cases [1]. Even with the considerable advances brought by targeted therapies and immunotherapy, survival remains limited—especially when the disease is diagnosed in its advanced stages, after metastasis has already occurred [2]. The presence of distant metastases plays a central role in shaping treatment options, influencing prognosis, and determining the overall clinical approach. Understanding how lung cancer spreads and what biological factors are linked to its metastatic behavior is essential for improving patient stratification and moving toward more individualized treatment strategies [3,4].

Metastatic patterns in lung cancer are far from uniform. While the liver, adrenal glands, brain, bones, and contralateral lung are frequently affected, the exact sites of spread can vary depending on the tumor histology, biological aggressiveness, and specific patient-related factors. Molecular profiling, such as the identification of Epidermal Growth Factor Receptor (EGFR), Anaplastic Lymphoma Kinase (ALK), or Kirsten Rat Sarcoma Viral Oncogene Homolog (KRAS) mutations, has become an indispensable tool for therapeutic guidance. Yet, despite its value, there is still a lack of simple easily accessible blood-based markers that can offer insights into how the disease might evolve and where it may metastasize [5,6,7].

Among the most accessible and routinely collected clinical data are complete blood count (CBC) parameters. These markers, often overlooked beyond their basic clinical utility, can reflect deeper systemic changes, including inflammation and the complex interplay between the tumor and the host’s immune system. Existing research has pointed to the prognostic relevance of markers like the neutrophil-to-lymphocyte ratio (NLR), absolute lymphocyte count, and platelet count in lung cancer [8,9]. However, their potential relationship with where metastases develop, especially when considered together with age, sex, tumor histology, or tumor location, has not been clearly established.

Many of the available studies have focused narrowly: either on the general prognostic impact of CBC markers or on a single metastatic site. What is missing is a more integrated view—one that combines laboratory, clinical, and pathological information to better understand metastatic tendencies. Given how widely available and low-cost CBC testing is it would be a missed opportunity not to explore its potential as a tool in predicting metastatic behavior [9,10,11].

Considering the variability in how lung cancer behaves from one patient to another, it makes sense to think that specific blood patterns might be associated with metastatic sites. For example, a high neutrophil count might reflect a more aggressive or immunosuppressive disease phenotype, possibly linked to liver or brain metastases, while higher lymphocyte levels could indicate a stronger immune environment, potentially influencing the pattern of spread [12,13].

So far, there has been no systematic exploration of these associations in the local patient population. Moreover, the influence of factors such as age and sex on these patterns remains insufficiently addressed.

The present study was developed to explore whether routinely measured blood parameters—including inflammation-related indices like NLR, PLR, and lymphocyte-to-monocyte ratio (LMR)—are linked to specific sites of metastasis in patients diagnosed with lung cancer. By combining comparative group analysis with logistic regression modeling, this study aims to highlight relevant hematological profiles that may offer practical value in anticipating metastatic trajectories and supporting more tailored clinical decisions.

## 2. Materials and Methods

### 2.1. Study Design and Setting

This study was conceived as a retrospective observational analysis carried out at a single center, with the goal of exploring how routine blood test parameters might relate to the anatomical distribution of metastases in patients diagnosed with lung cancer. The research took place at OncoHelp Medical Center in Timișoara, Romania, a specialized oncology facility that offers comprehensive cancer care, including diagnosis, systemic therapies, radiotherapy, and palliative support.

Patient data were gathered over a 36-month period, from 1 January 2022 to 31 December 2024. The retrospective design allowed for the inclusion of a clearly defined group of patients whose medical records and laboratory results were complete and available at the time of diagnosis. Because the data were collected in a real-life clinical setting, the findings reflect the complexity and diversity of routine oncology practice, making the results not only relevant but also applicable to everyday care.

The study population included patients from both urban and rural areas in Western Romania, offering a diverse and representative snapshot of those living with metastatic lung cancer in the region. This variability enriched the dataset and provided an opportunity to observe how biological and clinical features can differ across populations.

All steps related to data collection, anonymization, and analysis were conducted in accordance with institutional policies and ethical guidelines, ensuring both scientific integrity and respect for patient confidentiality.

### 2.2. Patient Selection

The study focused on adult patients (aged 18 years or older) with a confirmed diagnosis of lung cancer, established through histopathological examination. All patients had been evaluated and/or treated at OncoHelp Medical Center between January 2022 and December 2024. Eligible individuals were identified using the institution’s electronic medical record system, based on diagnostic codes specific to lung cancer.

To be included in the analysis, patients needed to meet the following criteria:

Age ≥ 18 years at the time of diagnosis;

- Histologically confirmed primary NSCLC [14];
- Radiological and/or histopathological confirmation of distant metastases present at the time of initial diagnosis, consistent with stage IV disease according to standard oncological staging systems (e.g., TNM classification);
- Availability of baseline hematological data, specifically a CBC, collected prior to the initiation of any oncologic treatment (chemotherapy, radiotherapy, or immunotherapy);
- Complete clinical documentation, including demographic information, tumor characteristics, and details regarding metastatic sites.

Certain patients were excluded in order to minimize confounding factors that could influence hematologic profiles independent of lung cancer. The exclusion criteria were as follows:

- Presence of concomitant hematologic malignancies such as leukemia, lymphoma, or myeloproliferative disorders, which could alter CBC values;
- Documented acute infections (bacterial, viral, or fungal) or systemic inflammatory diseases (e.g., autoimmune conditions, sepsis) at the time of blood testing;
- Use of chronic immunosuppressive therapies—including corticosteroids, biologics, or chemotherapy—prior to baseline hematologic assessment;
- Known severe bone marrow suppression caused by conditions unrelated to lung cancer, such as aplastic anemia or marrow infiltration from other malignancies.

### 2.3. Data Collection

The data analyzed in this study were collected retrospectively from the electronic medical records of patients treated at OncoHelp Medical Center in Timișoara. For everyone who met the inclusion criteria, relevant clinical, demographic, imaging, and laboratory information was carefully reviewed and extracted using a standardized protocol designed to maintain consistency and minimize selection or information bias.

The demographic and clinical variables included patient age at the time of diagnosis, sex, place of residence (urban or rural), and, when documented, exposure to known risk factors such as tobacco use, alcohol consumption, or occupational exposure to carcinogens. Tumor-related characteristics were also recorded, including histological subtype, the anatomical location of the primary tumor (classified as central or peripheral), and the clinical stage at diagnosis. Only patients with radiologically or histologically confirmed distant metastases involving a single anatomical site were selected for further analysis.

Information regarding metastatic involvement was obtained primarily from diagnostic imaging reports (CT, MRI, or PET-CT) and supplemented, when available, by biopsy or histopathology results. Metastases were classified according to their anatomical location, with a focus on the most frequently affected distant sites such as the pleura, contralateral lung, bone, liver, brain, and other less common locations including skin, adrenal glands, soft tissues, or peritoneum. To ensure clarity in interpreting hematological associations, the study included only those patients who presented with a single site of distant metastatic disease at the time of diagnosis.

Laboratory data consisted of CBC results collected at diagnosis, prior to the initiation of any systemic oncologic treatment. The parameters assessed included leukocyte, neutrophil, lymphocyte, monocyte, eosinophil, basophil, erythrocyte, and platelet counts. From these values, systemic inflammatory markers were calculated for each patient: NLR, PLR, and LMR, all of which have previously been proposed as potential indicators of the interaction between tumor biology and host immune response.

All collected data were anonymized and securely stored in a password-protected database. Each record was carefully verified for accuracy prior to statistical analysis, to ensure data quality and reliability.

### 2.4. Inflammatory Marker Calculation

To investigate the relationship between systemic inflammation and metastatic patterns, three widely recognized hematologic indices were calculated for each patient using standard formulas based on CBC data.

The NLR was obtained by dividing the absolute neutrophil count by the absolute lymphocyte count. This marker is commonly interpreted as a reflection of the balance between pro-inflammatory innate immune activation and the adaptive immune response [15].

The PLR was calculated by dividing the platelet count by the absolute lymphocyte count. PLR has been associated with cancer progression and tumor-related thrombocytosis, suggesting a link between inflammation, coagulation pathways, and tumor biology [16].

The LMR was determined by dividing the absolute lymphocyte count by the absolute monocyte count. This index is often used as an indicator of the host’s immune competence in contrast to the tumor-promoting role of monocytes [17].

All values were derived from baseline laboratory tests performed at the time of diagnosis, prior to the initiation of any oncologic treatment. These indices were analyzed as continuous variables and included in both comparative and regression models to assess their potential association with the presence and anatomical distribution of distant metastases.

### 2.5. Statistical Analysis

Statistical analyses were performed using GraphPad Prism version 9.0 (GraphPad Software, San Diego, CA, USA) and IBM SPSS Statistics version 26 (IBM Corp., Armonk, NY, USA). Continuous variables were expressed as the mean ± standard deviation (SD) and assessed for normality using the Shapiro–Wilk test. Between-group comparisons for normally distributed variables used the independent-samples *t*-test, whereas non-normally distributed data were analyzed using the Mann–Whitney U test.

For comparisons involving more than two subgroups, one-way ANOVA or Kruskal–Wallis tests were applied, depending on the data distribution. To control the family-wise error rate, statistical tests were organized into families of hypotheses, defined as sets of comparisons addressing the same biological question and involving the same inflammatory marker. Specifically:

- Family 1: all pairwise comparisons of NLR between metastatic sites.
- Family 2: all pairwise comparisons of PLR between metastatic sites.
- Family 3: all pairwise comparisons of LMR between metastatic sites.
- Family 4: logistic regression models testing associations between individual hematologic parameters and a specific metastatic location.

Multiple testing correction was applied independently within each family of hypotheses. For pairwise comparisons following ANOVA or Kruskal–Wallis tests, *p*-values were adjusted using the Benjamini–Hochberg false discovery rate (FDR) method, applied separately within each family of hypotheses (NLR, PLR, LMR) and within each group of hematologic parameters. Adjusted *p*-values are reported directly in the tables. Associations with FDR-adjusted *p* < 0.05 were considered statistically significant, and those between 0.05 and 0.10 were reported as trends. For logistic regression models, 99% confidence intervals (CIs) were reported to conservatively account for multiple testing. Bonferroni adjustment was used for post-hoc pairwise comparisons after ANOVA/Kruskal–Wallis tests, and 99% CIs were reported for logistic regression models to conservatively account for multiple testing within each family.

Odds ratios (ORs) for logistic regression were calculated using standardized z-scores to ensure comparability across variables. Effect sizes and observed statistical power were computed for each analysis. Unless otherwise specified, a *p*-value < 0.05 (two-tailed) was considered statistically significant after family-wise correction. For logistic regression models, raw and adjusted *p*-values (Holm) were calculated across all predictors tested, with statistical significance determined based on adjusted *p* < 0.05.

### 2.6. Ethical Considerations

This study was carried out in accordance with the ethical principles set forth in the Declaration of Helsinki and complied with all relevant institutional and regulatory guidelines. The research protocol received approval from the Ethics Committee of OncoHelp Medical Center, Timișoara (approval number 1186/7 May 2025).

Due to the retrospective design of the study, which involved the use of anonymized clinical and laboratory data obtained during standard medical care, the requirement for informed consent was formally waived by the Ethics Committee. All collected data were treated with strict confidentiality and stored in secure password-protected databases, accessible only to authorized members of the research team.

No experimental interventions were performed, and all procedures remained strictly within the boundaries of routine clinical practice. The authors confirm that no conflicts of interest were present and that patient privacy and data integrity were fully respected throughout the study.

## 3. Results

An overview of the baseline demographic, clinical, and pathological characteristics of the study population is provided to offer context for the subsequent analyses. Key variables such as patient age, sex distribution, and type of residence (urban or rural) are presented in Table 1. Relevant risk factors, such as occupational exposure to carcinogens, smoking history, and alcohol use, are also included where documented. In addition, details on histological subtypes, anatomical tumor location, and disease stage at the time of diagnosis are reported to illustrate the clinical profile of the cohort.

Descriptive statistics for key hematological parameters are presented in Table 2, including values for leukocytes, neutrophils, red blood cells, platelets, and various white blood cell subtypes. The data are expressed as means with standard deviations, along with observed minimum and maximum values, providing an overall view of the baseline hematologic profile of the study population.

The elevated mean levels of leukocytes and neutrophils, accompanied by wide standard deviations and high upper values, suggest the presence of systemic inflammation or possible infection in a subset of patients. The considerable variability observed in platelet counts may reflect tumor-associated thrombopoietic activity or an ongoing inflammatory response. Red blood cell levels appear relatively stable, while fluctuations in eosinophils, basophils, and monocytes may indicate varying degrees of immune activation across the cohort. Altogether, these findings highlight the biological heterogeneity of the study population and may carry prognostic or diagnostic relevance when interpreted alongside clinical data.

Table 3 offers an overview of the metastatic landscape in our study cohort. One of the first things that stands out is the uneven distribution of metastases: brain involvement is clearly the most prevalent, followed by bone metastases, while liver and pleural lesions appear far less frequently. This hierarchy mirrors the well-established tendency of solid tumors to preferentially colonize highly vascularized tissues that offer favorable immunological niches.

However, the breakdown of these four major metastatic sites is more than just descriptive. It has direct implications for the analyses that follow. The fact that roughly one-third of patients present with brain metastases provides solid statistical power to investigate hematologic markers associated with this pattern of spread. On the other hand, the relatively low number of pleural metastases serves as a cautionary note: any findings related to this group should be interpreted carefully due to limited sample size. Interestingly, the comparable proportions of bone and liver involvement give us a good opportunity to contrast the inflammatory profiles between these two sites without introducing significant sample imbalance.

This diverse distribution reflects the underlying biological heterogeneity of lung cancer and supports the stratified approach adopted throughout the rest of the study. In the following sections, the relationship between hematologic parameters, systemic inflammatory markers, and each metastatic pattern identified in Table 3 will be explored in detail.

Molecular profiling was available for 146 patients (48.3% of the cohort), performed according to standard clinical indications. The distribution of key molecular alterations is presented in Table 4. EGFR mutations were identified in 26 patients (17.8% of those tested), most frequently among patients with brain metastases (42.3% of EGFR-mutant cases; *p* = 0.028). KRAS mutations were detected in 36 patients (24.6%), with the highest proportion in those with liver metastases (38.9% of KRAS-mutant cases; *p* = 0.041). ALK rearrangements were rare (*n* = 8; 5.5%) but occurred exclusively in brain (62.5%) and pleural (37.5%) metastases. TP53 alterations were the most common genomic abnormality (*n* = 48; 32.8%), predominantly in bone (31.3%) and liver (27.1%) involvement. High PD-L1 expression (≥50%) was documented in 41 patients (28.1% of those tested) and was more frequent in pleural (29.3%) and brain (26.8%) metastases, without reaching statistical significance across sites (*p* = 0.271).

Statistical significance was evaluated using *p*-values adjusted for multiple testing (Bonferroni or Holm method, depending on data distribution), as shown in Table 5 and Table 6.

Comparative hematologic profiles across clinical sub-groups are presented in Table 5. PLE vs. non-PLE. Significant differences emerged for erythrocytes, eosinophils, and lymphocytes, all backed by very strong power (≈90–100%). Every other blood-cell line failed to reach significance, and, with observed power below 10%, we cannot confidently rule out subtle effects—though, practically speaking, any such effects are likely trivial.

OSS vs. non-OSS. The OSS subgroup showed higher total leukocytes, neutrophils, erythrocytes, eosinophils, and monocytes. Leukocytes and neutrophils met the classic 80% power threshold, while eosinophils were over-powered thanks to a large effect size. Erythrocytes and monocytes sit just below 80%; a handful of extra OSS cases would lock them in. Platelets, basophils, and lymphocytes showed no detectable differences—power for these tests never exceeded 18%.

HEP vs. non-HEP. HEP patients displayed significantly higher leukocytes, neutrophils, platelets, eosinophils, basophils, and lymphocytes. Leukocytes, neutrophils, eosinophils, and lymphocytes enjoyed excellent power (≥95%). Platelet power hovered around 63%, while basophils (≈62%) would benefit from a slightly larger HEP cohort. No meaningful signal appeared for erythrocytes or monocytes; power < 40% suggests any true effect is small at most.

BRA vs. non-BRA. Four variables stood out clearly—erythrocytes, eosinophils, basophils, and monocytes—each supported by ≥92% power. In contrast, leukocytes, neutrophils, platelets, and lymphocytes failed to hit significance, and their power was modest (<40% for the first two, <20% for platelets and lymphocytes). Detecting such small effects would require far larger BRA numbers, making a new recruitment push hard to justify.

For all pairwise comparisons, both raw and adjusted *p*-values are reported. Adjusted *p*-values were obtained using the Bonferroni correction for normally distributed variables (post-hoc after ANOVA) and the Holm method for non-normally distributed variables (post-hoc after Kruskal–Wallis). Adjusted values were used to determine statistical significance, with *p* < 0.05 considered significant after correction. This approach controls the type I error rate across multiple testing within each family of hypotheses.

For each comparison, Cohen’s d was calculated as a standardized effect size to quantify the magnitude of the differences between groups, complementing the adjusted *p*-values; values of 0.2, 0.5, and 0.8 were interpreted as small, moderate, and large effects, respectively.

Table 6 summarizes the three composite inflammation scores (NLR, PLR, and LMR) calculated for each metastatic subgroup and contrasted with the non-metastatic cohort, with both raw and adjusted *p*-values reported. Adjusted *p*-values were obtained using the Bonferroni correction or Holm method (depending on data distribution) to control for multiple testing within each family of hypotheses, and these adjusted values were used to determine statistical significance. The analysis offers a snapshot of how pro-inflammatory cells (neutrophils, platelets, monocytes) balance against the regulatory lymphocyte pool, depending on the organ involved.

Overall, the NLR was elevated across all metastatic sites, confirming that a systemic inflammatory tone accompanies disseminated disease. After adjustment for multiple comparisons, the increase reached statistical significance only for bone and liver metastases, pointing to a particularly neutrophil-driven milieu in these locations.

The PLR showed a more selective pattern: after *p*-value adjustment, a significant rise was observed exclusively in pleural metastases. This finding supports the notion that platelets may facilitate tumor cell anchoring and survival within the pleural cavity, without exerting the same influence in bone, liver, or brain.

Conversely, the LMR emerged as the most discriminating marker. Lower adjusted values characterized pleural and bone metastases, likely reflecting an expanded monocyte compartment, whereas patients with brain lesions displayed significantly higher adjusted LMR values, suggesting a relatively preserved or even heightened lymphocyte response in the cerebral setting.

**Figure 1:**
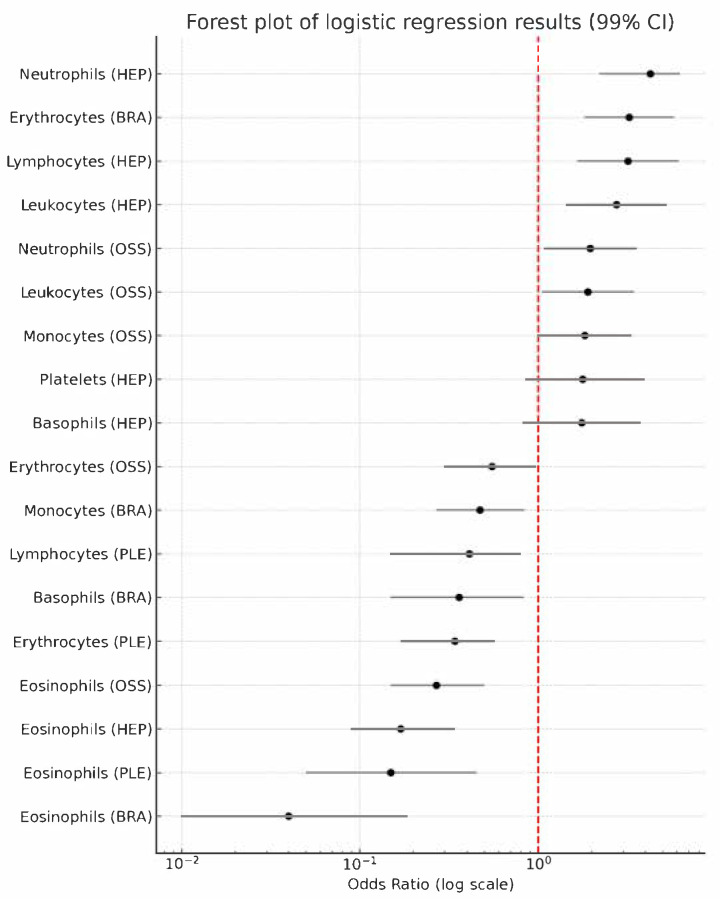
Forest plot of adjusted odds ratios (99% CI) for metastatic site predictors.

Table 7 and Figure 1 distils the single-marker logistic models linking each blood parameter to a specific metastatic niche. All predictors were z-standardized; so, the odds ratios (ORs) correspond to a one–standard-deviation increase, and we report 99% confidence intervals to keep only the most robust signals above the noise created by multiple testing.

For pleural spread, the pattern is almost anti-inflammatory: higher erythrocyte, eosinophil, and lymphocyte count each translate into a markedly lower probability of pleural involvement, and their 99% bands sit comfortably below unity. These associations remained significant after *p*-value adjustment.

In osteolytic bone lesions, the picture flips. Leukocytosis, neutrophilia, and monocytosis all push the odds upward, whereas a relative rise in erythrocytes or eosinophils seems protective. The protective effect of erythrocytes approached the threshold after adjustment, suggesting that a slightly larger OSS cohort could sharpen or soften this signal.

The hepatic lesions show the strongest systemic inflammatory signature of all sites. Total leukocytes, the neutrophil subset, and even lymphocytes are positively and convincingly associated with liver metastases, with all three retaining significances after adjustment. Platelets and basophils display only marginal links; their wide intervals betray limited precision and call for multivariable confirmation.

Finally, brain lesions carry their own fingerprint: relative polycythemia predicts a three-fold increase in risk, while eosinophils, basophils, and monocytes behave in the opposite direction. The inverse association with eosinophils is particularly striking, remaining significant after *p*-value adjustment and surviving the very conservative 99% threshold.

Inflammatory markers and metastatic patterns were analyzed across three age groups: 28–50, 51–70, and 71–78 years (Table 8). No statistically significant differences were observed in NLR, PLR, or LMR between groups (all ANOVA *p* > 0.1), though a trend toward higher NLR and PLR was noted with increasing age.

Regarding metastatic distribution, no significant age-based differences were found for lymphatic, pleural, pulmonary, hepatic, or other metastases. However, osseous metastases were significantly more frequent in the 28–50 age group compared to older groups, and brain metastases were markedly more prevalent in younger patients (28–50) versus older cohorts.

These findings suggest that while systemic inflammatory markers remain relatively stable with age, certain metastatic patterns, particularly osseous and cerebral, appear more common in younger patients.

## 4. Discussion

In just a few short years, low-grade systemic inflammation has gone from being considered mere biological background noise to an active player in the progression of solid tumors [18,19]. Particularly in non-small-cell lung cancer (NSCLC), elevated peripheral neutrophil levels and NLR have been consistently linked to poorer prognosis and increased metastatic potential [20,21]. The data presented in this study, drawn from a carefully selected cohort of NSCLC patients with a single metastatic site at diagnosis, suggest that the inflammatory ratios we routinely review in a standard blood count may, even subtly, offer clues about the organ most likely to be colonized by the tumor. In this context, the most consistent and robust signal emerged from neutrophils, underscoring their central role in metastatic dissemination [22]. Importantly, in this analysis, statistical significance was determined using *p*-values adjusted for multiple testing (Bonferroni or Holm method, depending on data distribution), and these adjusted values are reported in Table 5 and Table 6.

The most consistent signal, even after multiple testing correction, came from the neutrophils. Both the NLR and the absolute neutrophil count were substantially higher in patients with bone and liver metastases. For hepatic involvement, an increase of 1 × 10^3^ neutrophils/µL was associated with an approximately 10% rise in the likelihood of liver metastasis. Interestingly, NLR itself decreased in this context, suggesting that absolute neutrophilia, rather than the ratio, drives the hepatic inflammatory signal. Similar observations have been reported by Halazun in colorectal liver metastases and by Foerster in melanoma [23,24].

From a biological standpoint, the liver represents a tolerogenic environment, rich in resident myeloid cells [25]. In such a setting, neutrophils can expand without triggering a lymphocytic response, and their NETs (neutrophil extracellular traps) release proteases and chemokines that effectively prepare the hepatic “soil” for incoming tumor cells [26]. In bone, neutrophils activate the IL-6/STAT3 pathway and, through degranulation, expose the matrix to osteoclast, mediated remodeling, a mechanism elegantly described by Yan and colleagues and later validated by in vivo studies [27,28].

When metastasis was confined to the pleura, the PLR showed a subtle increase, after adjustment, remaining the only metastatic subgroup with this pattern. This aligns with previous findings in the literature, which have shown that platelets can envelop tumor emboli, shielding them from natural killer (NK) cells while delivering a concentrated supply of VEGF and TGF-β—key drivers of intrapleural angiogenesis [29].

The pleural environment, characterized by negative pressure and a mesothelial surface rich in podoplanin, appears to promote platelet aggregation [30]. One study previously described a similar phenomenon in the pathophysiology of pulmonary vessels [31]. The fact that PLR levels were not similarly elevated in liver or bone metastases suggests that platelet involvement is not merely a bulk inflammatory response but rather a targeted reaction shaped by the mechanical and molecular properties of each metastatic microenvironment.

The behavior of the LMR showed a mirrored pattern. After multiple testing correction, it remained significantly lower in patients with bone and pleural metastases, likely reflecting an expanded monocyte compartment, whereas patients with brain lesions showed a marked and significant increase, hinting at a relatively preserved or even heightened lymphocyte response in the cerebral setting. Once monocytes reach the bone marrow, they can be reprogrammed into tumor-associated macrophages (TAMs) with osteoclast-activating properties, leading to CCL2 and IL-10 production and promoting bone resorption [32].

In contrast, the brain, shielded by the blood–brain barrier, restricts monocyte infiltration [33]. Peripheral lymphocytes remain largely unaffected, while resident microglia take over the role of macrophages [34]. The elevated LMR observed in brain metastases aligns with findings from the Liu group, who reported colder immune profiles in cerebral lesions [35].

Some differences that were significant in the raw analysis did not retain significance after adjustment, highlighting the importance of controlling the type I error rate when performing multiple pairwise comparisons. This conservative approach strengthens confidence in the associations that remain, particularly the neutrophil-driven signals in bone and liver, the platelet-associated signal in pleura, and the LMR divergence between bone/pleura and brain metastases.

The molecular distribution in our cohort aligns with previously reported site-specific predilections in metastatic NSCLC. Molecular profiling was available for 146 patients (48.3% of the cohort), performed according to clinical indications and local testing availability. EGFR-mutant tumors showed a marked propensity for CNS dissemination, consistent with the literature suggesting enhanced survival and growth of EGFR-driven clones in the brain microenvironment, potentially facilitated by the blood–brain barrier’s immune modulation [36,37]. KRAS mutations were more frequent among patients with liver metastases, supporting existing evidence of a neutrophil-driven inflammatory niche that favors hepatic colonization, particularly in KRAS-mutant disease [38].

ALK rearrangements, although rare, were observed exclusively in patients with brain or pleural metastases, echoing the distinct metastatic patterns and high CNS tropism described in ALK-positive NSCLC [39]. TP53 alterations were common and associated with bone and liver involvement, in line with their established link to aggressive disease biology and poor prognosis [40].

High PD-L1 expression was detected across all sites, with a non-significant trend toward pleural and brain involvement, suggesting that immune checkpoint expression may be shaped more by tumor–host interactions than by metastatic location alone [41].

Integrating these molecular features with systemic inflammatory markers could improve our ability to stratify patients by metastatic risk. For instance, the co-occurrence of KRAS mutations with elevated neutrophil counts may identify a subgroup at particularly high risk for hepatic spread, while EGFR mutations coupled with higher LMR could help flag early brain involvement. Future studies should validate such combined profiles in prospective multicenter cohorts with comprehensive molecular and immune profiling.

In clinical settings, the CBC remains a widely accessible and low-cost investigation routinely performed in oncology patients. While not a substitute for imaging or molecular profiling, these blood-based markers may offer early indirect indications of metastatic spread. In resource-limited environments, such markers could aid in prioritizing diagnostic pathways and guiding clinical suspicion.

From a biological standpoint, the findings support the concept that cancer progression is not an isolated event but a dynamic interaction between tumor biology, systemic inflammation, and immune modulation [42]. Inflammation acts as both a driver and a reflection of metastatic potential, adapting in response to tumor location and immune resistance [43]. Understanding these relationships through routinely available laboratory data could bridge the gap between molecular insights and bedside decision-making.

While no single marker provides definitive predictive value, the observed patterns suggest that systemic inflammatory profiles may differ depending on metastatic site. These associations provide a deeper context for interpreting inflammatory responses in lung cancer and open the door for more personalized and integrated approaches to patient assessment.

The key innovation of this study lies in showing that simple inflammatory ratios extracted from a standard complete blood count—PLR, NLR, and LMR—yield distinct systemic signatures that can predict the metastatic destination (pleura, bone, liver, or brain) in lung-cancer patients with a single metastatic site. This discovery highlights the power of an inexpensive routine blood test to serve as a non-invasive triage tool for pinpointing early tumor spread, offering an innovative and accessible way to personalize the diagnostic pathway in oncology.

### Strengths, Limitations, and Future Directions

One of the major strengths of this study lies in its real-world applicability. By relying on routinely performed complete blood counts obtained during standard oncological care, the findings reflect practical clinical scenarios and emphasize the potential utility of these inexpensive and widely available markers in daily practice. The inclusion of a relatively large demographically diverse cohort—comprising patients from both urban and rural areas—enhances the external validity and generalizability of the results. Another important strength is the site-specific analytical approach: instead of pooling all metastases together, inflammatory markers were examined for each metastatic site separately. This stratified analysis provides a more nuanced understanding of tumor–host interactions and organ-specific inflammatory responses. Furthermore, this work is among the few to systematically investigate the variability of NLR, PLR, and LMR in relation to metastatic location in NSCLC, using logistic regression to explore their independent contributions to the systemic inflammatory profile. A further limitation is that molecular profiling was available for less than half of the cohort, which may have introduced selection bias and limited the statistical power to detect associations between specific genomic alterations and metastatic sites.

Despite these advantages, the study has several limitations. First, its retrospective single-center design may introduce selection bias and limits the generalizability of the findings to other populations and healthcare settings. Second, only a single baseline CBC measurement was available for analysis, precluding assessment of longitudinal trends or temporal changes in inflammatory markers before and after disease progression or treatment. Third, while exclusion criteria were applied to minimize confounding, residual confounders likely remain; for example, subclinical infections, undiagnosed inflammatory or autoimmune disorders, variations in nutritional status, smoking history, or use of non-oncological medications (such as corticosteroids, NSAIDs, or anticoagulants) could have influenced the CBC parameters. Fourth, other important prognostic and biological factors—such as molecular tumor profiles (EGFR, KRAS, ALK mutations), PD-L1 expression, or systemic inflammatory indices beyond NLR/PLR/LMR—were not incorporated into the analysis, potentially limiting mechanistic interpretation.

Additionally, the study excluded patients with multiple metastatic sites, which, while necessary for isolating site-specific signatures, may reduce the applicability to the broader stage IV NSCLC population. The lack of validation in an independent or prospective cohort further limits the strength of causal inference.

Another limitation of the study is the absence of a comparative group consisting of patients with the same histologic type of NSCLC but without metastases at the time of diagnosis. This lack of a control group prevents the adjustment of NLR, PLR, and LMR values to a baseline reference and may reduce the specificity of the observations. Our design, focused exclusively on stage IV patients with a single metastatic site, was chosen to minimize heterogeneity and to isolate organ-specific inflammatory profiles. Future studies should include non-metastatic comparison cohorts to validate and refine these findings.

Given the retrospective and exploratory nature of the study, the reported associations should be regarded as hypothesis-generating rather than confirmatory and require validation in independent prospective cohorts.

Future investigations should aim to validate these findings in larger multicenter cohorts with prospective designs. Serial measurement of inflammatory markers could help clarify whether changes in NLR, PLR, and LMR precede metastasis or reflect evolving disease burden. Integration of these markers with tumor genomics, imaging data, and clinical characteristics may lead to the development of predictive tools or composite risk scores for metastatic dissemination.

Further research should also examine whether these markers are associated with therapeutic outcomes, particularly in patients receiving immunotherapy. Identifying hematologic profiles that predict response to immune checkpoint inhibitors could optimize treatment selection. Additionally, mechanistic studies investigating how immune cell populations influence organ-specific metastatic patterns may shed light on the biological processes underlying the observed associations and help inform new therapeutic strategies.

## 5. Conclusions

This study explored the associations between systemic inflammatory markers derived from routine CBC and the anatomical distribution of distant metastases in patients with NSCLC. By focusing on a well-defined cohort of stage IV patients with a single metastatic site at diagnosis, the analysis provides targeted insights into how inflammation-related hematologic profiles may reflect underlying metastatic behavior.

Among the three evaluated markers—NLR, PLR, and LMR—distinct patterns emerged. Elevated neutrophil counts and NLR values were most strongly associated with bone and liver metastases, suggesting a neutrophil-driven inflammatory environment in these locations. In contrast, pleural metastases were characterized by a selective rise in PLR, pointing to a possible role for platelet-mediated mechanisms in intrapleural tumor dissemination. LMR showed a divergent trend, with lower values in bone and pleural metastases and higher levels in brain metastases, potentially reflecting site-specific immune dynamics.

Although none of the markers demonstrated sufficient discriminatory power to serve as standalone diagnostic tools, their organ-specific variations may offer supportive information in clinical decision-making—particularly in settings where access to advanced imaging is limited. These findings also reinforce the idea that systemic inflammation is not uniform across metastatic sites and may mirror the immune microenvironment of target organs.

These results should be interpreted as exploratory and hypothesis-generating, pending external validation.

## Figures and Tables

**Table 1:** Baseline clinical and demographic characteristics of the study cohort.

Characteristic	Value (*n* = 302)
Age (Mean ± SD)	60.71 ± 13.44
Sex	
Male	244 (80.79%)
Female	58 (19.20%)
Environment	
Urban	128 (42.38%)
Rural	174 (57.61%)
Occupational exposure to carcinogens	202 (66.88%)
Smoking or alcohol history	196 (64.90%)
Histological subtype	
Adenocarcinoma (ADK)	162 (53.64%)
Squamous cell carcinoma	110 (36.42%)
Other	30 (9.93%)

**Table 2:** Descriptive statistics of peripheral blood parameters in the cohort.

Parameter	Mean ± SD	Min	Max
Leukocytes (×10^3^/µL)	12.14 ± 8.39	1.71	56.00
Neutrophils (×10^3^/µL)	9.47 ± 7.69	1.02	52.00
Erythrocytes (×10^6^/µL)	4.24 ± 0.61	2.40	5.50
Platelets (×10^3^/µL)	369.66 ± 153.84	52.00	882.00
Eosinophils (×10^3^/µL)	0.36 ± 0.20	0.00	3.06
Basophils (×10^3^/µL)	0.03 ± 0.02	0.00	0.19
Monocytes (×10^3^/µL)	0.70 ± 0.44	0.05	3.00
Lymphocytes (×10^3^/µL)	1.58 ± 0.65	0.43	3.96

**Table 3:** Distribution of metastatic sites in the study cohort.

Metastasis Type	Number of Patients	Percentage (%)
PLE	52	17.21
OSS	86	28.47
HEP	66	21.85
BRA	98	32.45

This table presents the frequency and percentage of patients with metastases identified at specific anatomical sites. Abbreviations: PLE—pleura; OSS—bone; HEP—liver; BRA—brain.

**Table 4:** Molecular features by metastatic site.

Molecular Feature	Frequency in Tested Patients (%)	Most Frequent in Metastases to	*p*-Value
EGFR mutation	17.8 (*n* = 26)	Brain (42.3%)	0.028 *
KRAS mutation	24.6 (*n* = 36)	Liver (38.9%)	0.041 *
ALK rearrangement	5.5 (*n* = 8)	Brain (62.5%), Pleura (37.5%)	0.052
TP53 alteration	32.8 (*n* = 48)	Bone (31.3%), Liver (27.1%)	0.063
PD-L1 ≥ 50%	28.1 (*n* = 41)	Pleura (29.3%), Brain (26.8%)	0.271

* Significant *p* value.

**Table 5:** Comparative hematologic profiles across clinical sub-groups.

Group	Leukocytes (×10^3^)	Neutrophils (×10^3^)	Erythrocytes (×10^6^)	Platelets (×10^3^)	Eosinophils	Basophils	Monocytes	Lymphocytes
PLE (*n* = 52)	11.86 ± 8.31	9.26 ± 7.27	4.02 ± 0.62	376.14 ± 154.01	188.24 ± 153.16	42.76 ± 24.76	710.71 ± 511.69	1374.52 ± 549.47
Non-PLE (*n* = 281)	12.44 ± 8.58	9.71 ± 8.23	4.37 ± 0.59	362.50 ± 155.40	420.26 ± 228.92	37.63 ± 19.54	686.58 ± 346.15	1720.32 ± 730.98
Cohen’s d	−0.068	−0.056	−0.589	0.088	−1.06	0.251	0.064	−0.49
*p*-value (raw)	0.658	0.716	**0.0005**	0.568	**0.0005**	0.106	0.681	**0.003**
*p*-value (adjusted)	0.716	0.999	**0.002**	0.999	**0.002**	0.424	1.000	**0.012**
Observed power (%)	7	7	97	90	100	38	7	90
OSS (*n* = 86)	14.06 ± 11.73	11.33 ± 10.56	4.10 ± 0.67	397.12 ± 143.73	133.08 ± 100.14	33.46 ± 32.14	795.38 ± 561.28	1569.23 ± 725.48
non-OSS (*n* = 226)	11.21 ± 6.12	8.58 ± 5.76	4.30 ± 0.58	376.44 ± 158.07	317.04 ± 296.82	30.37 ± 27.69	652.96 ± 362.07	1581.48 ± 613.15
Cohen’s d	0.354	0.372	−0.33	0.134	−0.712	0.107	0.334	−0.019
*p*-value (raw)	**0.005**	**0.003**	**0.009**	0.290	**0.0005**	0.400	**0.008**	0.885
*p*-value (adjusted)	**0.013**	**0.120**	**0.036**	0.999	**0.002**	1.000	**0.032**	1.000
Observed power (%)	80	83	74	18	100	13	75	5
HEP (*n* = 66)	15.33 ± 12.84	14.21 ± 11.68	4.17 ± 0.74	405.33 ± 163.99	139.52 ± 113.16	35.24 ± 24.23	843.81 ± 585.80	1868.10 ± 768.16
non-HEP (*n* = 263)	11.00 ± 5.84	8.50 ± 5.46	4.26 ± 0.57	356.97 ± 149.00	299.15 ± 175.82	30.00 ± 14.44	747.80 ± 364.87	1474.07 ± 570.54
Cohen’s d	0.559	0.800	−0.148	0.318	−0.966	0.311	0.230	0.641
*p*-value (raw)	**0.0005**	**0.0005**	0.282	**0.021**	**0.0005**	**0.024**	0.096	**0.001**
*p*-value (adjusted)	**0.002**	**0.002**	0.999	**0.084**	**0.002**	0.096	0.384	**0.002**
Observed power (%)	98	100	19	63	100	62	38	100
BRA (*n* = 98)	13.34 ± 12.27	11.14 ± 13.67	4.58 ± 0.60	354.64 ± 112.47	86.00 ± 77.16	19.09 ± 17.58	553.64 ± 289.39	1534.55 ± 623.83
non-BRA (*n* = 204)	11.95 ± 7.18	9.21 ± 6.39	4.19 ± 0.60	372.06 ± 159.98	385.51 ± 136.06	35.01 ± 31.98	722.46 ± 454.91	1584.35 ± 654.97
Cohen’s d	0.152	0.206	0.650	−0.119	−2.491	−0.566	−0.413	−0.077
*p*-value (raw)	0.210	0.089	**0.0005**	0.331	**0.0001**	**0.0005**	**0.0005**	0.527
*p*-value (adjusted)	0.28	0.356	**0.002**	0.999	**0.002**	**0.002**	**0.002**	1.000
Observed power (%)	24	40	100	16	100	99	92	10

Abbreviations: PLE—pleura; OSS—bone; HEP—liver; BRA—brain; Displayed *p*-values are adjusted for multiple comparisons using the Bonferroni/Holm method within each family of hypotheses. Displayed *p*-values are adjusted for multiple comparisons using the Benjamini–Hochberg false discovery rate (FDR) method, applied within each group of comparisons. Significant values (FDR-adjusted *p* < 0.05) are in bold.

**Table 6:** Association between metastasis type and systemic inflammatory markers.

Metastasis Type	NLR ‡	PLR ‡	LMR ‡	*p*-Value (NLR)—Raw	*p*-Value (NLR)—Adjusted	*p*-Value (PLR)—Raw	*p*-Value (plr) —Adjusted	*p*-Value (LMR)—Raw	*p*-Value (LMR)—Adjusted
PLE	6.7	274	1.9	0.220	0.880	**0.006**	**0.0240**	**0.018**	0.072
OSS	7.2	253	2.0	**0.036**	0.144	0.410	1.000	**0.032**	**0.048**
HEP	7.6	217	2.2	**0.041**	0.164	0.150	0.600	0.300	1.000
BRA	7.3	231	2.8	0.150	0.600	0.810	1.000	**0.008**	**0.032**

Abbreviations: PLE—pleura; OSS—bone; HEP—liver; BRA—brain; ‡ NLR = neutrophil-to-lymphocyte ratio; PLR = platelet-to-lymphocyte ratio; LMR = lymphocyte-to-monocyte ratio. Note: *p*-values adjusted for multiple comparisons using the Benjamini–Hochberg false discovery rate (FDR) method, applied within each family of hypotheses. Significant values (FDR-adjusted *p* < 0.05) are in bold.

**Table 7:** Logistic regression analysis of inflammatory markers and hematologic parameters by metastasis type.

Metastasis Type	Marker	OR 99 †	*p*-Value Raw	*p*-Value Adjusted
PLE	Erythrocytes	0.34 (0.17–0.57)	0.001	0.018
	Eosinophils	0.15 (0.05–0.45)	0.001	0.018
	Lymphocytes	0.41 (0.15–0.80)	0.001	0.018
OSS	Leukocytes	1.90 (1.05–3.45)	0.006	0.018
	Neutrophils	1.96 (1.08–3.56)	0.004	0.072
	Erythrocytes	0.55 (0.30–0.97)	0.010	0.180
	Eosinophils	0.27 (0.15–0.50)	0.001	0.018
	Monocytes	1.83 (1.01–3.33)	0.009	0.162
HEP	Leukocytes	2.75 (1.44–5.28)	0.001	0.018
	Neutrophils	4.26 (2.20–6.25)	0.001	0.018
	Platelets	1.78 (0.85–3.72)	0.021	0.378
	Eosinophils	0.17 (0.09–0.34)	0.001	0.018
	Basophils	1.76 (0.82–3.77)	0.024	0.432
	Lymphocytes	3.20 (1.66–6.15)	0.001	0.018
BRA	Erythrocytes	3.25 (1.82–5.82)	0.001	0.018
	Eosinophils	0.04 (0.01–0.18)	0.001	0.018
	Basophils	0.36 (0.15–0.83)	0.001	0.018
	Monocytes	0.47 (0.27–0.84)	0.001	0.018

Logistic regression analysis by metastasis type. Odds ratios (OR) are for a one standard-deviation increase, with 99% CIs. Raw and adjusted *p*-values (Bonferroni and Holm) are shown; Abbreviations: PLE—pleura; OSS—bone; HEP—liver; BRA—brain. The label “OR 99 †” indicates that the reported odds ratios (OR) are expressed together with their 99% confidence intervals (99% CI). The dagger symbol (†) is used only as a footnote marker to clarify that all odds ratios are calculated per one–standard-deviation increase in the respective hematologic parameter.

**Table 8:** Comparative analysis of systemic inflammatory markers and metastasis distribution by age group.

	Age 28–50 (*n* = 35)	Age 51–70 (*n* = 206)	Age 71–78 (*n* = 61)	*p*-Value ANOVA	*p*-Value Age 28–50 vs. Age 51–70	*p*-Value Age 28–50 vs. Age 71–78	*p*-Value Age 51–70 vs. Age 71–78
Metastases PLE	4 (11.11%)	37 (18.97%)	11 (15.49%)	0.628	0.467	0.561	>0.999
Metastases OSS	11 (30.55%)	61 (31.28%)	14 (19.71%)	0.550	0.843	0.469	0.335
Metastases HEP	7 (19.44%)	44 (22.56%)	15 (21.21%)	0.832	>0.999	0.801	0.601
Metastases BRA	13 (36.11%)	64 (32.82%)	21 (29.57%)	0.726	0.557	0.827	0.641

## Data Availability

The data presented in this study are available upon request from the corresponding author. The data are not publicly available due to hospital policy.

## Abbreviations

The following abbreviations are used in this manuscript:

NSCLC	Non-small-cell lung cancer
CBC	Complete blood count
NLR	Neutrophil-to-lymphocyte ratio
PLR	Platelet-to-lymphocyte ratio
LMR	Lymphocyte-to-monocyte ratio
